# Exploring Epithelial–Mesenchymal Transition Signals in Endometriosis Diagnosis and In Vitro Fertilization Outcomes

**DOI:** 10.3390/biomedicines9111681

**Published:** 2021-11-12

**Authors:** Vito Cela, Elisa Malacarne, Maria Elena Rosa Obino, Ilaria Marzi, Francesca Papini, Francesca Vergine, Elena Pisacreta, Elisa Zappelli, Deborah Pietrobono, Giorgia Scarfò, Simona Daniele, Ferdinando Franzoni, Claudia Martini, Paolo Giovanni Artini

**Affiliations:** 1 Division of Gynecology and Obstetrics, Department of Clinical and Experimental Medicine, University of Pisa, 56126 Pisa, Italy; celav2001@gmail.com (V.C.); malacarne.eli@gmail.com (E.M.); mariaelena.obino@gmail.com (M.E.R.O.); ilaria.marzi1@gmail.com (I.M.); papinifra@libero.it (F.P.); fmvv@hotmail.it (F.V.); elepisacreta@gmail.com (E.P.); 2 Department of Pharmacy, University of Pisa, 56126 Pisa, Italy; eli.zap@hotmail.it (E.Z.); debh89@libero.it (D.P.); simona.daniele@unipi.it (S.D.); claudia.martini@unipi.it (C.M.); 3 Division of General Medicine, Department of Clinical and Experimental Medicine, University of Pisa, 56126 Pisa, Italy; giorgiascarfo91@gmail.com (G.S.); ferdinando.franzoni@unipi.it (F.F.)

**Keywords:** endometriosis, follicular fluid, inflammatory cytokines, EMT

## Abstract

Endometriosis (EMS) pathogenesis has been related to the release of inflammatory mediators in peritoneal fluid, creating an altered microenvironment that leads to low-grade oocyte/embryos and to the reduction of implantation rates. The Epithelial–Mesenchymal Transition (EMT), an inflammation-related process, can be a further contributing factor to EMS. This study aimed to investigate, among various cytokines and EMT markers (Cadherins, TGF-β, HIF-1α), diagnostic markers of EMS and prognostic factors of in vitro fertilization (IVF) outcomes. Herein, EMS patients manifested higher serum levels of the inflammatory molecules IL-6, IL-8, and IL-12 and a decrease in the concentrations of the anti-inflammatory IL-10. Moreover, biochemical markers associated with the EMT process were more elevated in serum and follicular fluid (FF) of EMS patients than in controls. At the end, the number of good-quality embryos was inversely related to serum IL-6 and EMT markers. Interestingly, serum IL-6 and FF IL-10 concentrations differentiated EMS patients from controls. Finally, serum IL-8 and E-Cadherin levels, as well as FF IL-10, predicted positive IVF outcome with great accuracy. Our data confirm the pivotal role of inflammatory mediators (i.e., IL-6 and IL-10) in EMS pathogenesis and suggest that EMT-related markers are elevated in EMS patients and can be predictive of IVF outcome.

## 1. Introduction

Endometriosis (EMS) is an estrogen-dependent chronic and progressive inflammatory disease that is characterized by the growth of endometrial-like tissue outside the uterine cavity [1]. The pathogenesis of these ectopic alterations lies in a multifactorial process that involves genetic and epigenetic factors, hormones, persistent peritoneal menstrual reflux, and exogenous factors [2]. Considering reproductive age, it affects 10–15% of all women and 30% of infertile women, with various symptoms including dyspareunia, dysmenorrhea, pelvic pain, headache, abdominal pain, and mood disorders [3]. 

An association between EMS and infertility has been widely reported in the literature, although the exact mechanism at the basis of this relationship is still a matter of debate, and explanations vary from anatomical anomalies (adhesions, fibrosis) and hormonal alterations to immunological processes [4]. In particular, morphological alterations and a decrease in cytoplasmic mitochondrial content impair oocyte quality in women affected by EMS [5]. Moreover, the presence of an ovarian EMS dramatically affects the ovarian reserve, since it seems to reduce the follicular density, the rate of follicles, and anti-Müllerian hormone (AMH) serum levels, related to the number of growing follicles [6]. Overall, EMS may affect the microenvironment of maturing oocytes, resulting in ovulatory dysfunction, poor oocyte quality, reduced fertilization rate, low-grade embryos, and reduced implantation rates, highlighting that EMS per se affects the development of oocytes and embryos and the receptivity of the endometrium [7]. Not surprisingly, recent meta-analyses suggest that patients with endometriosis-associated infertility undergoing in vitro fertilization (IVF) have a decrease in pregnancy rate as compared with other indications for IVF [7]. From a molecular point of view, endometrial infiltrations create a microenvironment characterized by increased oxidative stress and inflammation, which predisposes a person to fibrotic tissue formation [8]. Therefore, the immune system plays a crucial role in causing an inflammatory state that contributes to the maintenance of the disease and seems to be related to infertility, because it alters hormones, oocytes, and the endometrium [9]. Moreover, inflammatory system triggers oxidative stress in the peritoneal fluid, leading to an accumulation of prostaglandins; proteases; inflammatory cytokines, such as Interleukin-1 (IL-1), Interleukin-6 (IL-6), and Tumor Necrosis Factor α (TNF-α); and angiogenic cytokines such as Interleukin-8 (IL-8) and Vascular Endothelial Growth Factor (VEGF). A dysregulation in cytokine profile occurs in the follicular fluid (FF) as well [1]. In this sense, FF can reflect the metabolic and hormonal processes occurring in the microenvironment of maturing oocytes, thus containing biochemical components such as cytokines, chemokines, growth factors, and steroid hormones [7]. These inflammatory mediators, as a result of local synthesis in the ovaries and from blood plasma cytokines, modulate the processes of follicular growth, oocyte maturation/ovulation, steroidogenesis, embryo development, and pregnancy [7]. Local and systemic inflammation in EMS has been recently related to the epithelial-to-mesenchymal transition (EMT), by which epithelial cells lose their typical polarized organization, acquiring the motility of mesenchymal cells. This phenomenon is associated with the loss of E-cadherin expression (which connotes an epithelial cell phenotype) and an increase in N-cadherin (which connotes a mesenchymal cell phenotype) [10]. In general, EMT is a physiological mechanism that ensures tissue differentiation and regeneration during fetal and adult life [11]. Moreover, EMT has a key role in folliculogenesis and reparative mechanisms of the reproductive system [12]. Nevertheless, an inflammatory environment, characterized by an increased production of several cytokines, in particular Transforming Growth Factor β (TGF-β), TNF-α, and Interferon γ (IFN-γ) [13], causes an EMT dysregulation in the epithelial cells of the uterus and/or annexes [14]. In patients with EMS, serum and ectopic endometrium also contain elevated levels of Hypoxia-Inducible Factor 1α (HIF-1α) that contribute to triggering this process [15]. Therefore, an imbalanced EMT could be the basis for the development of pathological processes such as adenomyosis, cancer, metastasis, and EMS [14]. In particular, it has been demonstrated that the EMT contributes to perpetuating endometrial lesions, causing an irritation that greatly impairs an eventual embryo implantation and a consequent successful IVF outcome [16]. 

Herein, patients with a clinical diagnosis of EMS and control women undergoing IVF were enrolled in the present study. Serum and follicular fluid (FF) were collected to measure inflammatory molecules and signals typically related to the EMT process (i.e, markers N-cadherin, E-cadherin, TGF-β, HIF-1α). The study’s goals were to find putative alterations related to inflammation and EMT process in serum and FF, correlate them to clinical parameters related to IVF outcome, and individuate diagnostic markers able to distinguish EMS patients from control women.

## 2. Materials and Methods

### 2.1. Patients

In this preliminary prospective study, we have compared clinical characteristic IVF outcomes and biological parameters between N = 32 patients with pelvic EMS and N = 32 patients with tubal and male factor infertility identified as controls, all of whom underwent IVF treatment at the Centre of Infertility and Assisted Reproduction of the Department of Clinical and Experimental Medicine of Pisa, between October 2018 and December 2019. 

All patients underwent a complete clinical history and physical examination, biochemical analyses, and transvaginal ultrasonography (US). Age, height, weight and body mass index (BMI) were also recorded. Fertility investigations included hysterosalpingography, hysteroscopy, cycle day 3 measurements of serum levels of estradiol (E2), follicle-stimulating hormone (FSH), anti-Müllerian hormone (AMH), transvaginal US with antral follicle count (AFC), and semen analysis for the partner. Infertile patients with EMS plus other associated dysfunctions, such as polycystic ovary syndrome, hyperprolactinaemia, thyroid dysfunction, hypothalamic amenorrhoea, Cushing’s syndrome, and congenital adrenal hyperplasia, were excluded from the study. In each patient, possible history of previous surgery was investigated. EMS patients enrolled in the study were comparable for medical history, ultrasound features, and history of infertility. All women had been proven infertile for at least 1 year. 

### 2.2. IVF Procedures

Patients were monitored and managed according to institutional clinical protocols. Controlled Ovarian Hyperstimulation (COH) was performed using 150–450 IU/day of recombinant FSH or hMG (human menopausal gonadotropin) in a gonadotropin-releasing hormone (GnRH) antagonist protocol (or long agonist protocol). The gonadotropin starting dose and type of COS protocol were established according to the characteristics of each patient (age, BMI, AFC, and AMH level). To inhibit premature LH surge, a daily dose of 0.25 mg of (GnRH) antagonist was administered with cetrorelix (Cetrotide, MerckSeronoSpa, Rome, Italy), on the base of personalized regimen, when the lead follicle reached 12–14 mm in diameter. 

Triggering of final oocyte maturation was carried out using 250 mg of recombinant hCG (Ovitrelle^®^, Merck Serono Europe Ltd., London, UK) or a GnRH analogous (Triptorelin 0.2 mg s.c, Decapetyl; Ipsen Spa, Milan, Italy) when at least 1–2 follicles had reached a mean diameter of 17 mm. At approximately 36 h after triggering, transvaginal follicular aspiration was performed for oocyte retrieval. Volume, sperm count, forward motility, and morphology were considered according to the World Health Organization criteria [17]. Oocytes collected by vaginal US were incubated in oocyte culture medium (Sydney IVF Oocyte Wash Buffer; Cook Ireland Ltd., Limerick, Ireland). IVF or ICSI were performed as appropriate, depending on semen quality and the patient’s clinical history. Four to five hours after oocyte retrieval, in cases where IVF was performed, each oocyte was inseminated with 200,000–300,000 motile washed spermatozoa, while ICSI was performed as described elsewhere [18]. Fertilization was confirmed by the observation of two pronuclei 16–18 h after the fertilization technique. All the fertilized oocytes were transferred into a fresh cleavage medium (Sydney IVF Cleavage Medium; Cook Medical, Bloomington, CA, USA) and cultured until embryo transfer (ET). On day 2, at 46–48 h post-insemination, the embryos were evaluated for cell number and rate of fragmentation, and consequently graded as I–IV (best to worst) by expert embryologists. ET was performed on day 2 or 5 under the guidance of abdominal US, using a K-Soft 500 Embryo Transfer Catheter^®^ (Cook Medical, Bloomington, CA, USA). From the day of the ET, all patients started luteal phase supported with vaginal micronized progesterone, 200 mg three times a day (Prometrium^®^, Rottapharm S.p.A., Milan, Italy), and intramuscular hydroxyprogesterone caproate every 72 h (Lentogest^®^, IBSA, Milan, Italy). In patients who underwent triggering with GnRH analogous, all oocytes and/or embryos obtained were vitrified. 

At the time of ovum pick-up, a blood sample was taken for each patient enrolled in the study with a 9 mL test tube. Some “pure” follicular fluid was also taken from at least one follicle, that is, before any use of the culture media.

### 2.3. Collection of Follicular Fluid Samples and Serum Samples

Follicular fluid from single follicles was obtained from 64 women by fine-needle aspiration on the day of oocyte retrieval, centrifuged at 4 °C for 10 min at 2500 rpm (to remove the cellular component), and stored at −80 °C until assayed. The serum was obtained by centrifugation at 4 °C (2500 rpm for 15 min) from blood samples taken on the day of the pick-up and stored at −80 °C until assay.

### 2.4. NFκb, N-Cadherin, and E-Cadherin Measurement in Follicular Fluid and Serum

The concentrations of p65 NFκb, N-cadherin, and E-cadherin in follicular fluid and serum samples were measured by an enzyme-linked immunosorbent assay (ELISA), as described [19,20,21]. Briefly, the plates were precoated with a specific antibody to p65 NFκb, N-cadherin, or E-cadherin, diluted in poly-L-ornithine, and maintained overnight at 4 °C. After washing with PBS-Tween 0.01%, BSA 1% was added for 2 h at 37 °C, to block the nonspecific sites. Follicular fluid or serum samples were added to each well for 2 h at 25 °C. Then, polyclonal antibodies to p65 NFκb (sc-372, Santa Cruz Biotechnology, anti-rabbit), N-cadherin (sc-7939, Santa Cruz Biotechnology, Dallas, TX, USA, anti-rabbit), or E-cadherin (#3195, Cell Signaling, anti-rabbit) were employed and incubated for 2 h at 25 °C. Subsequently, a specific HRP antibody (Santa Cruz Biotechnology, Dallas, TX, USA) was added to each well and incubated for 1 h at 37 °C. The 3,3′,5,5′-tetramethylbenzidine (TMB) (Thermo Fisher Scientific, Waltham, MA, USA) and, consequently, a stop solution (H_2_SO_4_) was added, and the absorbance was read at 450 nm (EnSight Multimode Plate Reader, PerkinElmer, Milan, Italy). All measurements were performed in triplicate, and a calibration line was performed for each protein.

### 2.5. Interleukines (ILs) and TGF-b Measurement in Follicular Fluid and Serum

TGF-β and all interleukins’ levels were measured using enzyme-linked immunosorbent assay (ELISA) kits (Cloud-Clone Corp., Katy, TX, USA: SEA124Hu-96 for TGF-β, SEA079Hu for IL-6, SEA080Hu for IL-8, SEA111Hu for IL-12, and SEA056Hu for IL-10) following the manufacturers’ instructions. Briefly, 100 µL of follicular fluid or serum (diluted 1:10 in Standard Diluent) and of each dilution of standard was added into the appropriate wells and incubated for 1 h at 37 °C. After the incubation time, 100 µL of primary antibody were added for 1 h at 37 °C. After extensive washes, 100 µL of secondary antibody were incubated for 30 min at 37 °C, and then the substrate solution was added to each well, leaving the color to develop for 10–20 min at 37 °C. Absorbance was measured at 450 nm. 

### 2.6. HIF-1α Measurement in Follicular Fluid and Serum

HIF-1α was determined using enzyme-linked immunosorbent assay (ELISA) kit (RAB1057-1KT, SigmaAldrich, Milan, Italy) following the manufacturers’ instructions. Briefly, 100 µL of each standard and samples were added into appropriate wells and incubated for 2.5 h at room temperature with gentle shaking. After extensive washes, wells were incubated with 100 µL of Biotinylated Detection Antibody for 1 h at room temperature and then with 100 µL of HRP-Streptavidin solution for 45 min. After incubation time, the TMB reagent was added for 30 min, and absorbance was measured at 450 nm. 

### 2.7. Statistical Analysis 

The GraphPad Prism (GraphPad Software Inc., San Diego, CA, USA) was used for data analysis and graphical presentations. All data are presented as the mean ± SEM. Statistical analysis was performed by a one-way analysis of variance (ANOVA) with Bonferroni’s corrected *t*-test for post hoc pair-wise comparisons. *p* < 0.05 was considered as statistically significant. (Mann–Whitney unpaired *t*-test). Correlation among variables was determined by linear regression analysis, while interactions among variables were calculated by correlation and multiple regression analyses. Covariate analysis was performed by the z test. All statistical procedures were performed using the StatView program (Abacus Concepts, Inc., SAS Institute, Cary, NC, USA) [22]. 

### 2.8. ROC Analysis

When significant differences had been detected at post hoc tests, the diagnostic potential of each biomarker was assessed by calculating the area under the receiver operating characteristic curve (AUROC) and its associated confidence intervals (CI) [23]. NCSS package version 2 was used; the statistical significance threshold level was set at *p* < 0.05. An AUC between 1 and 0.8000 indicates an excellent accuracy of the biomarker; 0.7900 < AUC < 0.6495 individuates a good biomarker. An interval from 0.6401 to 0.6494 indicates a discreet or fairly good marker. AUC values lower than 0.6400 identify a poor accuracy. 

## 3. Results

### 3.1. Descriptive Statistics 

The main clinical characteristics and IVF outcome parameters of EMS patients and controls are shown in Table 1. Mean age, BMI, and levels of FSH, LH, and E2 were found to be comparable among the groups. 

A statistical analysis was performed to compare the clinical data reported in Table 1 for EMS and control women. Patients with endometriosis presented lower AFC (*p* = 0.0006) and AMH (*p* = 0.1596) levels, although the latter did not reach significant value (see discussion section). 

Number of follicles > 16 mm at the trigger day (*p* = 0.003), total number of oocytes retrieved (*p* = 0.0055), number of MII oocytes (*p* = 0.0136), and fertilization rate (*p* = 0.0494) were significantly lower in women with EMS than in controls. 

### 3.2. Covariate Analyses of Basal and IVF Clinical Parameters

A multivariate analysis was performed on basal clinical parameters of the total population. BMI negatively affected AMH levels (*p* = 0.0145) and was inversely related to both LH (*p* = 0.0064) and FSH (*p* = 0.0474) levels, suggesting that body mass is actually linked to fertility-related parameters. As expected, age negatively influenced AMH levels in the total population (*p* = 0.0274). Furthermore, AMH values were strictly related to AFC (*p* < 0.0001) and negatively related to FSH. On the other hand, LH was positively related to FSH levels (*p* < 0.0001).

In summary, AMH levels were confirmed to be strictly related to AFC (*p* < 0.0001) and to be decreased by increasing age, BMI, and FSH levels.

Of note, AMH negative correlations with BMI (*p* = 0.8914) or age (*p* = 0.592) were completely lost in patients with EMS, thus confirming that the pathology affects ovarian reserve per se. Consistently, BMI showed an inverse correlation with AFC in the control group only (*p* = 0.0004) and a stronger negative relation with AMH (*p* < 0.0001) in controls than in the patients’ group. AMH and AFC were directly related to most of the examined IVF-related parameters, i.e., total units of gonadotropins (AMH: *p* = 0.0023; AFC: *p* = 0.0265), days of stimulation (AMH: *p* = 0.0279; AFC: *p* = 0.0022), E2 at the trigger day (AMH: *p* < 0.001; AFC: *p* = 0.007), number of follicles >16 mm at the trigger day (AMH: *p* = 0.0251; AFC: *p* = 0.0439), total number of oocytes retrieved (AMH: *p* < 0.001; AFC: *p* = 0.0054), number of MII oocytes (AMH: *p* < 0.001; AFC: *p* = 0.0112), fertilization rate (AMH: *p* = 0.0008; AFC: *p* = 0.0405), and number of good-quality embryos (AMH: *p* = 0.0216; AFC: *p* = 0.0225).

In contrast, FSH levels were inversely related to E2 at the trigger day (*p* = 0.0006), the number of follicles >16 mm at the trigger day (*p* = 0.0438), total number of oocytes retrieved (*p* = 0.004), number of MII oocytes (*p* = 0.0012), and fertilization rate (*p* = 0.0054) and directly related to progesterone level at the trigger day (*p* = 0.0362). 

As expected, women’s age correlated with total units of gonadotropins (*p* = 0.0156). Interestingly, BMI was directly related to both total units of gonadotropins (*p* < 0.001) and days of stimulation (*p* = 0.0043). 

### 3.3. Interleukin’s Concentrations in Serum and Follicular Fluid of Control and Patients with EMS

A high concentration of cytokines has been found in serum of patients with EMS, implying that EMS could result in systemic inflammation. However, it is unclear whether the inflammation is a consequence of EMS [24,25]. The amount of selected inflammatory and anti-inflammatory ILs was measured in serum of control and EMS women (Table 2). 

**Figure 1:**
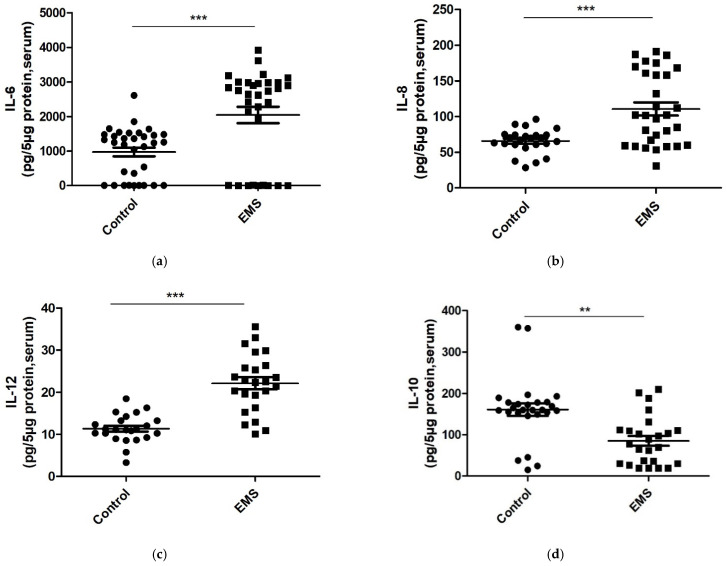
Protein levels of proinflammatory IL-6 (**a**), IL-8 (**b**), IL-12 (**c**), and anti-inflammatory IL-10 (**d**) in serum samples from control women and EMS patients. All interleukins were quantified using commercial ELISA kits (Cloud-Clone ELISA kit). The data are reported as the mean values ± SD of three independent experiments, each performed in duplicate. Statistical analysis was performed by unpaired *t*-test: ** *p* < 0.01, *** *p* < 0.001 versus control.

In our study, patients with EMS showed significantly higher serum concentrations of the proinflammatory cytokines IL-6 (*p* < 0.0001, Figure 1a), IL-8 (*p* = 0.0002, Figure 1b, Table 2), and IL-12 (*p* < 0.0001, Figure 1c, Table 2) than in controls. 

Consistent with these data, patients recruited for the study presented a significant decrease in the levels of the anti-inflammatory cytokine IL-10 (*p* = 0.0041, Figure 1d, Table 2) as compared to the control group.

**Figure 2:**
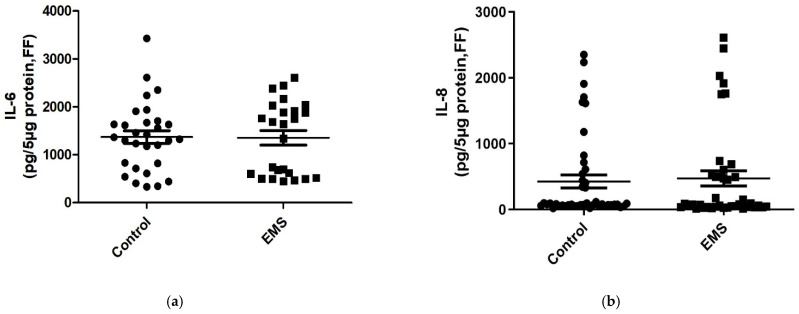

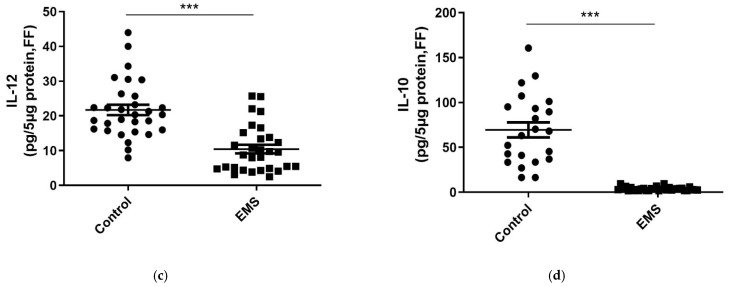
Protein levels of proinflammatory IL-6 (**a**), IL-8 (**b**), IL-12 (**c**), and anti-inflammatory IL-10 (**d**) in follicular fluid (FF) samples from control women and EMS patients. The data are reported as the mean values ± SD of three independent experiments each performed in duplicate. Statistical analysis was performed by unpaired *t*-test: *** *p* < 0.001 versus control.

Next, in order to examine if the inflammatory alterations can be revealed in biological fluids other than serum, the aforementioned interleukins were examined in follicular fluid as well (Table 3). As depicted in Figure 2a,b, (Table 3), comparable levels of IL-6 (*p* = 0.3908) and IL-8 (*p* = 0.2524) were noticed in follicular fluid of patients with EMS and controls. In contrast, as revealed in serum, significantly lower levels of the anti-inflammatory cytokine IL-10 were detected in FF of EMS patients compared to controls (*p* < 0.0001, Figure 2d, Table 3). 

Surprisingly, IL-12 concentration in FF of patients with endometriosis was significantly lower than in control women (*p* < 0.0001, Figure 2c, Table 3). In our hand, IL-12 concentration in serum and FF showed an opposite trend in patients and control women.

### 3.4. Evaluation of EMT Markers in Serum and Follicular Fluid of Control and Patients with Endometriosis

The putative association of EMT with EMS and its correlation with IVF-related parameters was evaluated by examining the concentrations of EMT-related biological markers (Table 2 and Table 3) 

**Figure 3:**
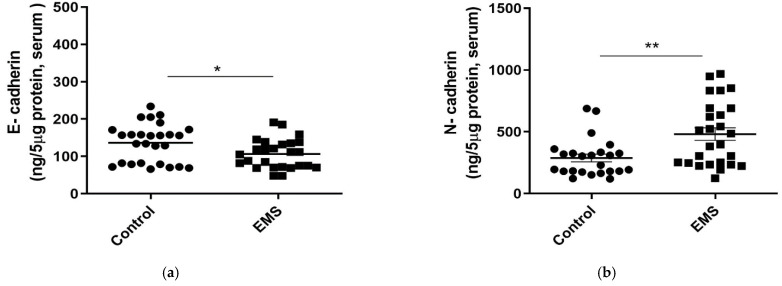

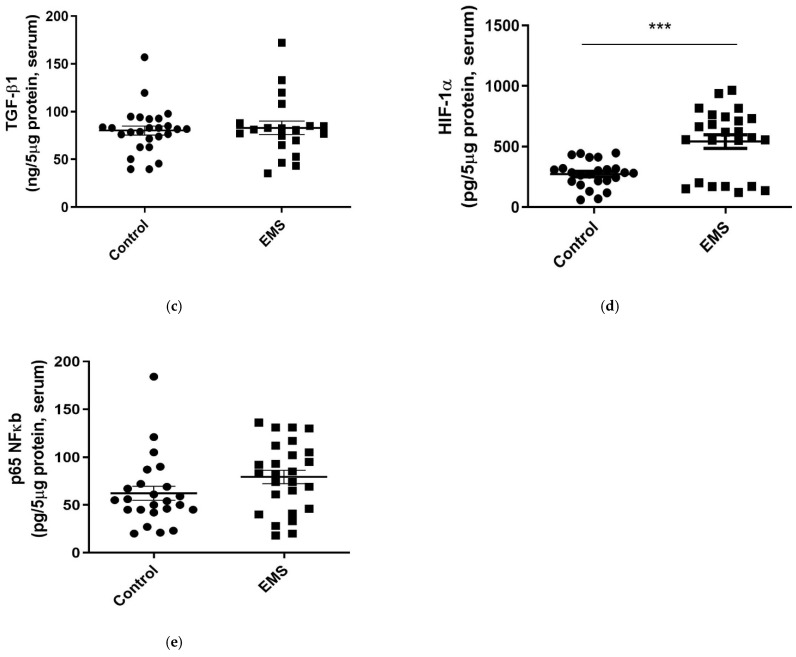
Evaluation of EMT markers (E-cadherin, N-cadherin) and EMT-related inflammatory markers (TGF-β, HIF-1α, and NF-kB) in serum samples from control and EMS patients (Endo). E-cadherin (**a**), N-cadherin (**b**), TGF-β (**c**), HIF-1α (**d**), and NF-kB (**e**) levels were assessed by homemade ELISA kits. Data are the mean ± SD of two different experiments, each performed in duplicate. Statistical analysis was performed by unpaired *t*-test: * *p* < 0.05, ** *p* < 0.01, *** *p* < 0.001 versus control.

The serum levels of the epithelial marker E-cadherin decreased significantly in patients with EMS with respect to controls (*p* = 0.02628, Figure 3a). Consistently, a significant increase in the levels of the mesenchymal marker N-cadherin was noticed in serum (*p* = 0.0087, Figure 3b). 

TGF-β, the major promoter of the EMT process, showed comparable levels in serum between patients with EMS and control women (Figure 3c, *p* = 0.4650). 

Nevertheless, HIF-1α, whose stability and expression are typically induced by TGF-β, was found significantly higher in serum of patients than in controls (*p* < 0.0001, Figure 3d). In addition, significantly higher levels of p65 NF-kB, typically involved in the regulation of EMT genes [26], did not change in the serum of patients compared to control group (Figure 3e, *p* = 0.1060). 

**Figure 4:**
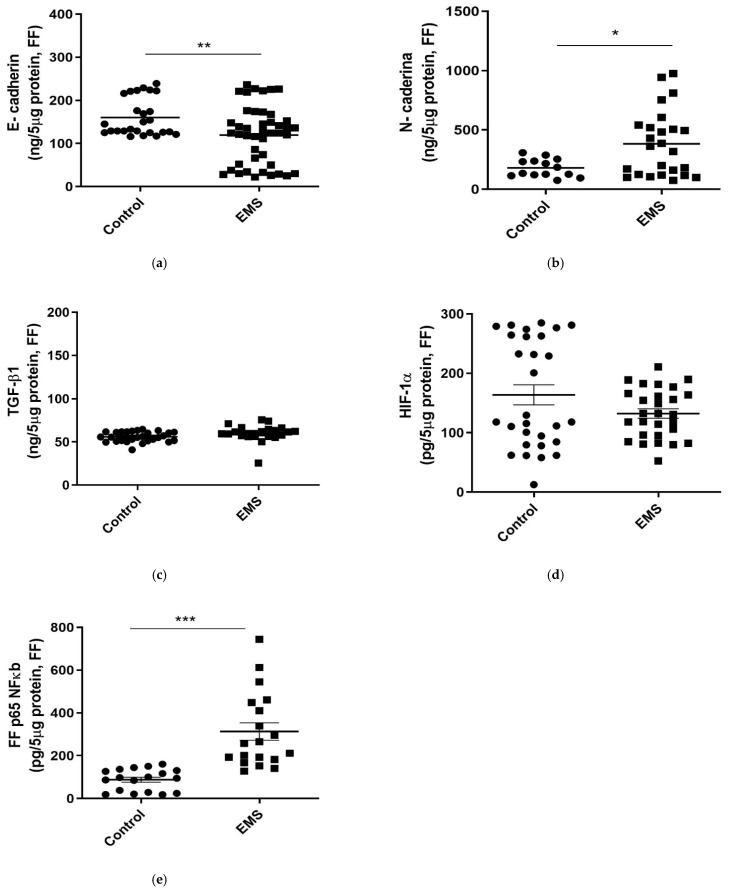
Evaluation of EMT markers (E-cadherin, N-cadherin) and EMT-related inflammatory markers (TGF-β, HIF-1α, and NF-kB) in FF samples from control and EMS patients. E-cadherin (**a**), N-cadherin (**b**), TGF-β (**c**), HIF-1α (**d**), and NF-kB (e) levels were assessed by homemade ELISA kits. Data are the mean ± SD of two different experiments, each performed in duplicate. Statistical analysis was performed by unpaired *t*-test: * *p* < 0.05, ** *p* < 0.01, *** *p* < 0.001 versus control.

As evidenced in serum, FF of patients with EMS was characterized by significantly lower concentrations of the epithelial marker E-cadherin (*p* = 0.0015, Figure 4a) and by an increase in the levels of the mesenchymal marker N-cadherin (*p* = 0.0134, Figure 4b). In contrast, comparable FF concentrations of TGF-β and HIF-1α were evidenced in the two groups of women (*p* = 0.5839 and *p* = 0.4650, respectively; Figure 4c,d).

Finally, FF concentrations of p65 NF-kB were found to be significantly increased in patients with respect to the control group (Figure 4e, *p* = 0.0010). 

### 3.5. Correlation Analyses of Biochemical Parameters

Serum IL-6 concentrations were positively related to the levels of IL-8 (*p* = 0.0215) and IL-12 (*p* = 0.0009) and inversely related to IL-10 ones (*p* = 0.0172). Similarly, serum IL-12 levels were directly related to the serum concentration of IL-8 (*p* = 0.0234) and negatively related to IL-10 ones (*p* = 0.0089). Interestingly, N-cadherin was positively correlated to NF-kB concentration (*p* = 0.00145) and inversely related to IL-10 (*p* = 0.0181) in serum. Furthermore, IL-6 levels in serum were inversely related to FF concentrations of IL-10 (*p* < 0.0001). Finally, TGF-β levels were positively correlated with IL-6 levels in FF (*p* < 0.0001). 

### 3.6. Correlation Analyses of Clinical and Biochemical Parameters

All the biochemical markers measured in serum and follicular fluid were correlated with the clinical results by linear regression analysis. 

IL-6 serum concentrations were inversely related to E2 level (*p* = 0.0371), the number of follicles >16 mm (*p* = 0.0007), oocytes retrieved (*p* = 0.0039), MII mature oocytes (*p* = 0.0052), and good-quality embryos (*p* = 0.0327). Of note, the number of follicles >16 mm was negatively related to serum IL-8 (*p* = 0.0315) as well as IL-12 (*p* = 0.0401) levels. 

**Figure 5:**
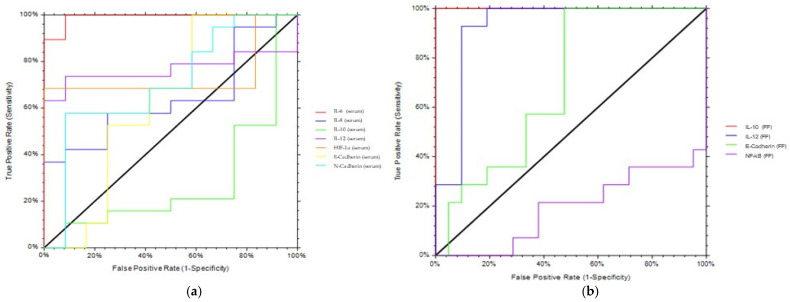

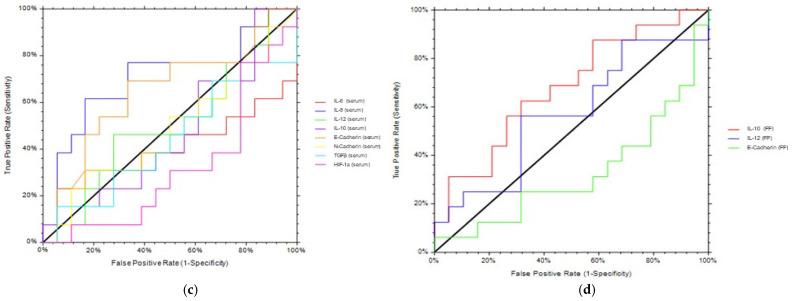
ROC analyses. ROC analyses of serum (**a**,**c**) and FF (**b**,**d**) parameters were performed to assess the ability to discriminate EMS patients from controls or to predict IVF outcome. AUROC, area under the receiving operating characteristic curve, and CI, confidence interval, are shown in Table 4.

Furthermore, the IL-8 levels in the serum were inversely correlated with AMH value in the total population (Figure 5a, *p* = 0.0277, R^2^ = 0.092). When the control subgroup was considered, serum IL-12 concentrations were positively related to FSH value (Figure 5b, *p* = 0.0189, R^2^ = 0.236). 

Consistent with its higher concentration in the control group, FF IL-12 concentration was positively related to the number of oocytes retrieved (*p* = 0.0275).

Concerning EMT biomarkers, the number of MII oocytes and good-quality embryos was inversely related to the serum concentration of NF-kB (MII oocytes: *p* = 0.0481; good-quality embryos: *p* = 0.0490, R^2^ = 0.106) and positively related to E-cadherin (MII oocytes: *p* = 0.0315; good-quality embryos: *p* = 0.0055, R^2^ = 0.150). 

### 3.7. ROC Analysis

For the significant differences detected in post hoc tests, the diagnostic potential of each biomarker was verified by calculating the AUROC and its associated CI [23]. The accuracy of serum and FF biomarkers in discriminating patients with endometriosis from control is summarized in Table 4 and Figure 5. Serum IL-6 demonstrated an excellent capacity in discriminating EMS patients from controls (AUROC = 0.994, Figure 5a); serum IL-12, HIF-1α, and N-cadherin displayed quite good accuracy (Figure 5a and Table 4), whereas IL-8 and E-cadherin in serum did not differentiate efficiently the two groups (Table 4). 

Concerning FF biomarkers, FF IL-10 discriminated perfectly ENDO patients from controls (AUROC = 1, Figure 5b); E-cadherin was shown to distinguish ENDO from controls with good accuracy (*p* = 0.6939). 

Next, we evaluated the putative efficacy of the aforementioned biomarkers in predicting IVF outcome (i.e., a positive value of β-hcg). Serum IL-8 and E-Cadherin, as well as FF IL-10, predicted positive values of β-hcg with quite great accuracy. All the other serum and FF biomarkers were fairly predictive of IVF outcome.

## 4. Discussion

The present study aimed to investigate, among various inflammatory factors, key serum and intrafollicular markers to differentiate control women from EMS patients and to predict good-quality oocytes/embryos in women undergoing IVF. The main findings of our study are as follows: (i) EMS patients presented significantly higher serum levels of the inflammatory molecules IL-6, IL-8, and IL-12 and a decreased concentration of the anti-inflammatory marker IL-10 compared to control women; (ii) biochemical markers associated with the EMT process (i.e, N-cadherin, NF-kB, TGF-β, and HIF-1α) were significantly more elevated in serum and/or FF of EMS patients than in control women. Our data confirm the pivotal role of inflammatory mediators in EMS and evidence the involvement of EMT process in its pathogenesis; (iii) IL-6 serum concentrations were inversely related to E2 level, the number of follicles >16 mm, oocytes retrieved, MII mature oocytes, and good-quality embryos; (iv) the number of follicles >16 mm was negatively related to serum IL-8 and IL-12 levels; (v) the numbers of MII oocytes and good-quality embryos were inversely related to the serum concentration of NF-kB and positively related to E-cadherin; (vi) serum IL-6 and FF IL-10 demonstrated an excellent capacity in discriminating EMS patients from controls. At the end, serum IL-8 and E-Cadherin, as well as FF IL-10, predicted positive values of β-hcg with quite great accuracy. These findings evidenced that IL-8, IL-10, and the EMT-related marker E-cadherin can be predictive of mature oocytes, good-quality embryos, and IVF outcome.

EMS pathogenesis and progression has been related to the release in peritoneal fluid of inflammatory mediators, including chemokines and cytokines, which modify the patients’ immune system and create an altered microenvironment that leads to ovulatory dysfunction, poor oocyte quality, low-grade embryos, and reduced implantation rates [8].

In recent years, the role of inflammation as a mechanism underlying the onset of endometriosis and consequent infertility has been discussed, especially with regards to EMT [27]. During EMT, epithelial cells lose the typical polarized organization, acquiring the motility and invasive features of mesenchymal cells, and it has been widely studied in oncological processes as regards the invasion of cancer cells and metastasis [28,29,30]. The molecular mechanisms characterizing EMT include the loss of some epithelial markers (including E-cadherin, desmoplankin, occludin, and so on), gaining mesenchymal markers (including N-cadherin, vimentin, and so on), resulting in significant changes in cellular activities such as subsequent ability to migrate, invasiveness, and resistance to apoptosis [14,31]. Starting from these assumptions, research on EMT implication in EMS has increasingly expanded, with the aim of determining its characteristics and finding possible therapeutic targets [32]. 

In this study, possible parameters of inflammation and the EMT process were analyzed in serum and FF of patients with EMS and in control women undergoing IVF.

In the cohort enrolled in the present study, EMS patients presented significantly lower AFC and levels of the clinical parameters related to IVF outcome than in control women, including the number of follicles >16 mm at the trigger day, total number of oocytes retrieved, number of MII oocytes, and fertilization rate. As expected, AMH levels were negatively influenced by age and FSH levels.

Of note, BMI negatively affected AMH levels and was inversely related to both LH and FSH levels, suggesting that body mass is actually linked to fertility-related parameters. Consistent with our data, recent studies have highlighted that ovarian reserve markers of AMH and FSH are significantly lower in obese than in nonobese women, and BMI is negatively correlated with AMH [33,34].

The observed relationship between BMI and AMH can explain the comparable levels of AMH between EMS patients and controls detected in this study: indeed, although it did not reach statistical significance (*p* = 0.1569), control women presented a higher mean of BMI (control women: 23.9 ± 5.6; EMS patients: 22.2 ± 3.7), which may have negatively affected AMH. Furthermore, BMI was observed to be directly related to both total units of gonadotropins and days of stimulation in the total population of enrolled women. Consistent with our results, Marci and coworkers have evidenced a significantly higher dosage of gonadotropins in patients with a BMI ≥ 25 kg/m², when compared to those with a normal BMI [35].

Interestingly, the AMH negative correlations with BMI or age were completely lost in EMS patients, thus suggesting that the pathology affects ovarian reserve per se.

Next, pivotal inflammatory mediators were measured in serum, collected at the trigger day, of the enrolled women. In our hands, EMS patients showed significantly higher serum concentrations of the proinflammatory cytokines IL-6, IL-8, and IL-12 with respect to controls. Among our results, other studies have confirmed an increase in serum IL-6 [36], IL-8, or IL-12 concentration [7].

Moreover, patients recruited for the study presented a significant decrease in the levels of the anti-inflammatory cytokine IL-10, as compared to the control group. Overall, our data confirm that EMS induces a systemic inflammatory reaction.

Of note, IL-6 serum concentrations were inversely related to E2 level, the number of follicles >16 mm, oocytes retrieved, MII mature oocytes, and good-quality embryos. The number of follicles >16 mm was negatively related to serum IL-8 and IL-12 levels. In this sense, recent meta-analyses support the association between elevated serum IL-6 and/or IL-8 concentrations and the occurrence of EMS-associated infertility [37].

Next, the aforementioned interleukins were also examined in FF, in order to determine if the inflammatory alterations can be revealed in other biological fluids than serum. The follicular fluid is considered as a biological “window” that reflects the hormonal and metabolic processes that contribute to the development of a suitable microenvironment for oocyte maturation and ovulation. The follicular fluid has been demonstrated to contain cytokines, chemokines, growth factors, angiogenic factors, and other essential elements for the folliculogenesis/oogenesis processes, creating a peculiar microenvironment that could negatively affect IVF outcomes [7,38,39]. In this sense, recent studies have detected higher follicular fluid levels of IL-8 and IL-6 [7,40], suggesting a local production or recruitment. In actuality, herein, comparable levels of IL-6 and IL-8 were noticed in FF of EMS patients and controls. Consistent with our paper, Tambo and co-workers have found comparable FF IL-6 and IL-8 levels between women with endometriomas and their control group [40], thus evidencing that EMS partially affect the FF status and composition.

Surprisingly, IL-12 concentration in FF of EMS patients was significantly lower with respect to control women. Thus, in our study, IL-12 concentration showed an opposite trend in serum and FF of patients and control women. Conflicting data have been reported on IL-12 in FF. For example, a recent study has reported a higher IL-12 level in FF of EMS patients [41] and has revealed IL-12 as a promising prognostic marker of oocyte and embryo quality in women with EMS. In contrast, IL-12 has been reported to positively correlate with oocyte fertilization and embryo development [42], as well as to play an important role in oocytes’ maturation. [43] Consistent with the latter data, herein, FF IL-12 concentration was positively related to the number of oocytes retrieved. We may speculate that IL-12 can play a positive role in the FF microenvironment, favoring oocytes’ development. On the other hand, its altered presence in serum can be a further sign of systemic inflammation. Further studies will elucidate this issue. 

Then, EMT-related signals were examined in the same cohort of women. The serum levels of the epithelial marker E-cadherin decreased significantly in patients with EMS with respect to controls, together with a consistent significant increase in the levels of the mesenchymal marker N-cadherin. Of note, comparable differences were observed in FF, thus suggesting that the EMT process is involved in both systemic inflammation and alteration of the local follicular microenvironment. Consistent with our data, E-cadherin levels have been shown to be lower in EMS patients [44]. Furthermore, another study has shown that epithelial cells of endometriotic lesions may have, at least in part, a higher level of mesenchymal features than normal endometrium [45].

The TGF-β signaling pathway seems to play an essential role in the EMT mechanism. For example, a TGF-β up-regulation has been highlighted in the endometriotic tissue, serum, and peritoneal fluid of EMS patients, demonstrating its possible role in the development and maintenance of this disease [32,46,47]. In our study, comparable levels of TGF-β were found in serum of EMS patients and control women. However, HIF-1α, whose stability and expression are typically induced by TGF-β, was found significantly increased in serum of patients compared to controls, thus confirming the fundamental role of hypoxia in endometriotic lesions [48].

Furthermore, significantly higher levels of p65 NF-kB, typically involved in the regulation of EMT genes [26], were found in FF of EMS patients with respect to the control group. Of note, the numbers of MII oocytes and good-quality embryos were inversely related to the serum concentration of NF-kB and positively related to E-cadherin, thus suggesting an involvement of the EMT process in IVF outcome too. Interestingly, the numbers of MII oocytes and good-quality embryos were inversely related to the serum concentration of NF-kB and positively related to E-cadherin. These data confirm that the transition to a mesenchymal phenotype may exacerbate a pre-existing altered microenvironment that invalidates IVF-related parameters.

Overall, our data evidenced a significant enhancement of inflammatory molecules and a decrease in anti-inflammatory ones in serum of EMS patients with respect to controls. Moreover, the analyzed biochemical markers associated with the EMT process were significantly higher in serum and/or FF of EMS patients than in control women, and the numbers of MII oocytes and good-quality embryos were inversely related to the serum concentration of parameters related to a mesenchymal transition. 

Further investigations will be needed to understand how (and by what potential mechanism) the EMT process may influence oocytes and embryos’ quality, and thus IVF outcome. In adult organisms, EMT occurs as a physiological response to injury during wound healing after ovulation [11,42] and regulates endometrial composition and regeneration under normal conditions, such as embryo implantation and the menstrual cycle [11]. However, dysfunction of EMT in the normal epithelial cells of the reproductive organs has been related to pathological processes, including EMS [11,44]. During EMT, epithelial cells lose their polarity and cell-to-cell adhesion properties, acquiring migratory characteristics related to migration and invasion. Numerous signaling pathways, transcription factors, and epigenetic modifiers [11] have been demonstrated to act in the local microenvironment to reprogram epithelial cells toward a more mesenchymal-like fate during EMT, resulting in an enhancement of local and systemic inflammation. An inflamed microenvironment may be at the basis of altered processes of folliculogenesis and embryo implantation. In this sense, the use of an IVF animal model may help to clarify the molecular mechanisms linking EMT and IVF outcome. 

For the significant differences detected at post hoc tests, the diagnostic potential of each biomarker was verified by calculating the AUROC and its associated CI. Serum IL-6 demonstrated an excellent capacity in discriminating EMS patients from controls (AUROC = 0.994); serum IL-12 and HIF-1α displayed good accuracy, whereas IL-8, N-cadherin and E-cadherin in serum poorly differentiated the two groups. Consistent with our data, IL-6 has been shown to discriminate between patients with endometriosis and controls [49,50]. 

Concerning potential FF biomarkers, FF IL-10 discriminated perfectly EMS patients from controls (AUROC = 1), thus evidencing the importance of a good local microenvironment for assuring IVF success. 

Next, we evaluated the putative efficacy of the aforementioned biomarkers in predicting IVF outcome (i.e, a positive value of β-hcg). Serum IL-8 and E-Cadherin, as well as FF IL-10, predicted β-hcg outcome with quite good accuracy, thus confirming that the inflammatory status strongly influences IVF procedures. 

To the best of our knowledge, serum and FF biochemical parameters related to the EMT process were related to IVF outcome for the first time. A recent paper has shown EMT induction in infertile patients is affected by chronic endometritis [16], thus underlying the role of EMT in inflammatory-related gynecological pathologies.

Overall, our data confirm the pivotal role of inflammatory mediators, such as IL-6 and IL-10, in EMS pathogenesis and suggest that these inflammatory indicators and the EMT-related marker E-cadherin can be predictive of mature oocytes, good-quality embryos, and IVF outcome. 

## Figures and Tables

**Table 1:** Descriptive statistics of clinical parameters for control women and patients with endometriosis. The data are expressed as mean ± SD. Statistical analysis was performed by unpaired *t*-test. * *p* < 0.05, ** *p* < 0.01, *** *p* < 0.001 vs. control women.

Parameter	Control (MEAN ± SD)	Endometriosis Patients (MEAN ± SD)
Age	34.9 ± 3.5	36.3 ± 3.9
BMI	23.9 ± 5.6	22.2 ± 3.7
FSH	7.78 ± 2.05	9.31 ± 3.85
LH	5.97 ± 2.56	5.61 ± 2.53
Estradiol	43.8 ± 27.4	49.4 ± 19.0
AMH	2.77 ± 1.31	2.14 ± 2.00
AFC	12.8 ± 4.8	8.68 ± 3.33 ***
Male age	37.8 ± 3.7	39.2 ± 3.7
Total units of gonadotropins	3254 ± 1573	3026 ± 759
Days of stimulation	10.0 ± 2.6	9.10 ± 1.86
Estradiol (trigger day)	2585 ± 1762	1793 ± 1687
Progesterone (trigger day)	1.44 ± 0.80	1.29 ± 0.81
Number of follicles >16 (trigger day)	6.27 ± 3.40	3.53 ± 1.81 **
Number of oocytes collected	5.50 ± 2.48	3.84 ± 2.08 **
Number of mature oocytes MII	4.47 ± 1.89	3.29 ± 1.79 *
Good-quality embryos	1.59 ± 0.80	2.79 ± 1.92
Number of fertilized oocytes	3.81 ± 2.04	1.19 ± 1.14 *

**Table 2:** Biochemical parameters measured in serum collected at the trigger day from control women and patients with endometriosis. Interleukin (IL) IL-6, IL-8, IL-10, HIF-1α (Hypoxia-inducible factor 1-alpha), and NFκB (Nuclear Factor kappa-light-chain-enhancer of activated B cells) are expressed as pg/5 ug total proteins. IL-12, TGF-β (Transforming growth factor beta), E-cadherin, and N-cadherin are expressed as ng/5 ug total proteins. All the data are expressed as mean ± SD. Statistical analysis was performed by unpaired *t*-test. * *p* < 0.05, ** *p* < 0.01, *** *p* < 0.001 vs. control women.

Serum Parameters	Control (MEAN ± SD)	Endometriosis Patients (MEAN ± SD)
IL-6	1335 ± 462	2842 ± 443 ***
IL-8	67.4 ± 18.6	110.7 ± 51.2 ***
IL-10	161.3 ± 77.9	85.3 ± 58.9 **
IL-12	11.36 ± 3.37	22.1 ± 6.9 ***
HIF-1α	272 ± 84	540 ± 222 ***
TGF-β	1625 ± 482	1660 ± 592
E-cadherin	137.9 ± 94.5	107.6 ± 31.9 *
N-cadherin	286.7 ± 118.6	525.3 ± 214.8 **
NFκB	64.1 ± 24.5	77.82 ± 29.1

**Table 3:** Biochemical parameters measured in FF, collected at the pick-up day, from control women and patients with endometriosis. IL-6, IL-8, IL-10, HIF-1α, and NFκB are expressed as pg/5 ug total proteins. IL-12, TGF-β, E-cadherin, and N-cadherin are expressed as ng/5 ug total proteins. All the data are expressed as mean ± SD. Statistical analysis was performed by unpaired *t*-test. * *p* < 0.05, ** *p* < 0.01, *** *p* < 0.001 vs. control women.

Follicular Fluid Parameters	Control (MEAN ± SD)	Endometriosis Patients (MEAN ± SD)
IL-6	1371 ± 716	1354 ± 754
IL-8	67.9 ± 21.5	57.8 ± 39.5
IL-10	87.7 ± 62.9	3.85 ± 2.06 ***
IL-12	21.7 ± 8.3	10.4 ± 6.7 ***
HIF-1α	174.4 ± 81.8	127.22 ± 35.3
TGF-β	1120 ± 117	1162 ± 362
E-cadherin	162.3 ± 39.1	141.8 ± 42.3 **
N-cadherin	183.1 ± 57.7	432.4 ± 253.3 *
NFκB	88.0 ± 42.6	312.9 ± 144.1 ***

**Table 4:** Diagnostic accuracies of serum and FF biomarkers in differentiating control women from EMS patients. AUROC, area under the receiving operating characteristic curve; CI, confidence interval.

GROUP COMPARISONS	PREDICTORS	AUC	95% CI
Controls vs. EMS patients	Serum IL-6	0.9912	0.9084–0.9992
	Serum IL-8	0.6447	0.4005–0.8034
	Serum IL-10	0.2807	0.0699–0.4675
	Serum IL-12	0.7675	0.5187–0.8965
	Serum HI1- α	0.7368	0.4804–0.8772
	Serum E-cadherin	0.6272	0.3298–0.8114
	Serum N-cadherin	0.7061	0.4455–0.8564
	FF IL-10	1.0000	
	FF IL-12	0.9252	0.7436–0.9797
	FF E-cadherin	0.6939	0.4717–0.8332
	FF NFkB	0.1905	0.0278–0.3433
β-hcg positive vs. β-hcg negative	Serum IL-6	0.4017	0.1473–0.6063
	Serum IL-8	0.7094	0.4460–0.8597
	Serum IL-12	0.5000	0.2554–0.6845
	Serum IL-10	0.4701	0.2308–0.6557
	Serum E-cadherin	0.6496	0.3916–0.8129
	Serum N-cadherin	0.4850	0.2394–0.6723
	Serum TGF-β	0.4402	0.1996–0.6307
	Serum HI1- α	0.3205	0.1126–0.5015
	FF IL-10	0.6776	0.4532–0.8213
	FF E-cadherin	0.5691	0.3366–0.7362
	FF NFkB	0.3257	0.1262–0.4998

## Data Availability

Data are contained within the article.
